# Dental age assessment of 3–15‐year‐old Saudi children and adolescents using Demirjian's method—A radiographic study

**DOI:** 10.1002/cre2.186

**Published:** 2019-04-29

**Authors:** Ahmed Alassiry, Khalid Alshomrani, Saeed Al Hasi, Ahmed Albasri, Saleh Said Alkhathami, Malak Abdulmajeed Althobaiti

**Affiliations:** ^1^ Faculty of Dentistry Najran University Najran Saudi Arabia; ^2^ Pediatric Dentistry Consultant in Prince Sultan Military Medical City Riyadh Saudi Arabia; ^3^ Orthodontist Private Centre Dammam Saudi Arabia; ^4^ Orthodontic Department Resident‐Saudi board in orthodontic Jeddah Saudi Arabia; ^5^ General Dentist Taif Saudi Arabia

**Keywords:** Alassiry, DEMIRJIAN’S method, Saudi Arabia

## Abstract

Dental age assessment plays a pivotal role in clinical practice, demographic studies, forensics, and courts of law but is affected by ethnic and geographic variations. The aim was to determine the population‐specific weighted scores needed when dental age is estimated using Demirjian's method for Saudi children and adolescents between the ages of 3 and 15 years. Design: A total of 298 panoramic radiographs were collected from Saudi Arabia. Dental age was assessed using Demirjian's method (1973). Chronological age was determined from the date of birth and the date on which the panoramic radiograph was taken for each individual. Between 3‐ and 15‐years age group, the Saudi boys had an estimated age of 9.07 ± 1.96 years and chronological age of 8.49 ± 2.30 years. The Saudi girls had an estimated age of 9.22 ± 1.93 years and chronological age of 8.78 ± 2.32 years. With Demirjian's method, the Saudi boys were 0.57 ± 1.48 years, and girls were 0.44 ± 1.66 years ahead of their chronological ages (*p* < .05). New population‐specific weighted scores were developed to convert dental age according to Demirjian's method into estimated ages in the contemporary Saudi Arabian population. This study can be used for further research and comparisons with other population groups, regions or communities.

## 1 INTRODUCTION

Age is a case of mind over matter. This philosophical saying may hold true in various walks of life but does not do well in medicine and dentistry. Age does matter, and it plays a vital role in the diagnosis and treatment planning of pedodontic and orthodontic patients. It has a defined role in the fields of forensic dentistry, endocrinology, orthopedics, demographics, and anthropology (Schmeling, Geserick, Reisinger, & Olze, 2007). Estimation of an individual's age may be important in criminal matters, assistance with illegal immigration in the absence of proper documents, identification of the ages of dead persons in natural calamities and disasters, and decisions regarding when a person can seek employment, marry, or go to prison (Bagic, Sever, Brkic, & Kern, 2008).

There are different types of age systems that can be used to measure the age of an individual. Numerical age, chronological age, skeletal age, dental age, developmental age, physiological age, biological age, bone age, mental age, and social age are some of them (Smith & Brownlees, 2011). Age is affected by gender, race, ethnicity, and the nutritional and endocrine status of an individual. Chronological age is a poor indicator of skeletal maturity because of significant individual variation (Bhanat & Patel, 2013). Dental age is considered to be a more reliable indicator of biological development and maturity in children as it is less affected by nutritional and endocrine status (McKenna, James, Taylor, & Townsend, 2002).

Dental age estimation can be done by morphological, biochemical, and radiological methods (Stavrianos, Mastagas, Stavrianou, & Karaiskou, 2008). Morphological methods are based on the ex vivo microscopic evaluation of extracted teeth. Biochemical methods involve the racemization of aspartic amino acids from dental tissues. Radiological methods of age assessment involve the study of timing and sequence of the eruption of teeth along with the degree of calcification of the set traits of teeth from radiographs. Calcification of teeth is a more reliable indicator when assessing dental age compared with the eruption sequence as it is more genetically determined and less governed by factors such as arch length discrepancy, loss of primary teeth, ankylosis, gingival thickness, and so forth. (Ogodescu, Bratu, Tudor, & Ogodescu, 2011). Many methods/scores and charts have been developed for age estimation in children and adolescents. Some of them include the Schour and Masseler method (1941), Nolla's method (1960), Moorees, Fanning, and Hunt method (1963), Cameriere method, Harris and Nortje method, van Heerden method, and Demirjian, Goldstein, and Tanner method (1973; Panchbhai, 2011). The method described by Demirjian et al. (Demirjian, Goldstein, & Tanner, 1973) in 1973 based on French‐Canadian children is the most widely used method due to its accuracy and feasibility. Many studies have used this method for comparisons with their population groups.

In Saudi Arabia, studies have been performed to establish age estimation using Demirjian's method on samples of individuals from middle and western regions. However, until now, no studies have been done to establish population‐specific weighted scores for forensic age estimation comprising all five regions of Saudi Arabia (northern, western, eastern, southern, and middle). The purpose of the study was to determine the population‐specific weighted scores needed when dental age is estimated using Demirjian's method for Saudi children and adolescents between 3 and 15 years of age.

## 2 MATERIAL AND METHODS

### 2.1 Materials

A total of 298 digital panoramic radiographs of children and adolescents in the age group of 3–15 years were collected from all five regions of Saudi Arabia. The sample consisted of 150 boys and 148 girls who attended dental clinics as a part of routine dental treatment. None of the digital panoramic radiographs were taken primarily for this research project. The records were sorted according to the region, age, and gender of the individuals. The study protocol was approved by the ethics committee of the Najran University.

### 2.2 Methods

#### 2.2.1 Inclusion criteria

Availability of date of birth and date of panoramic radiograph;
Good quality radiographs;
Full complement of mandibular teeth present (erupted or unerupted);
No history of orthodontic treatment;
Absence of systemic diseases;
Non‐syndromic patients; and
No abnormal dental problems such as congenitally missing teeth, ankylosis, or impaction.

#### 2.2.2 Exclusion criteria

Incomplete patient records;
Poor quality of diagnostic radiographs;
Patient with congenital anomalies such as cleft lip and palate, aplasia, hypodontia, supernumerary teeth, and impaction;
Extraction of permanent teeth; or
History of trauma.

**Figure 1:**
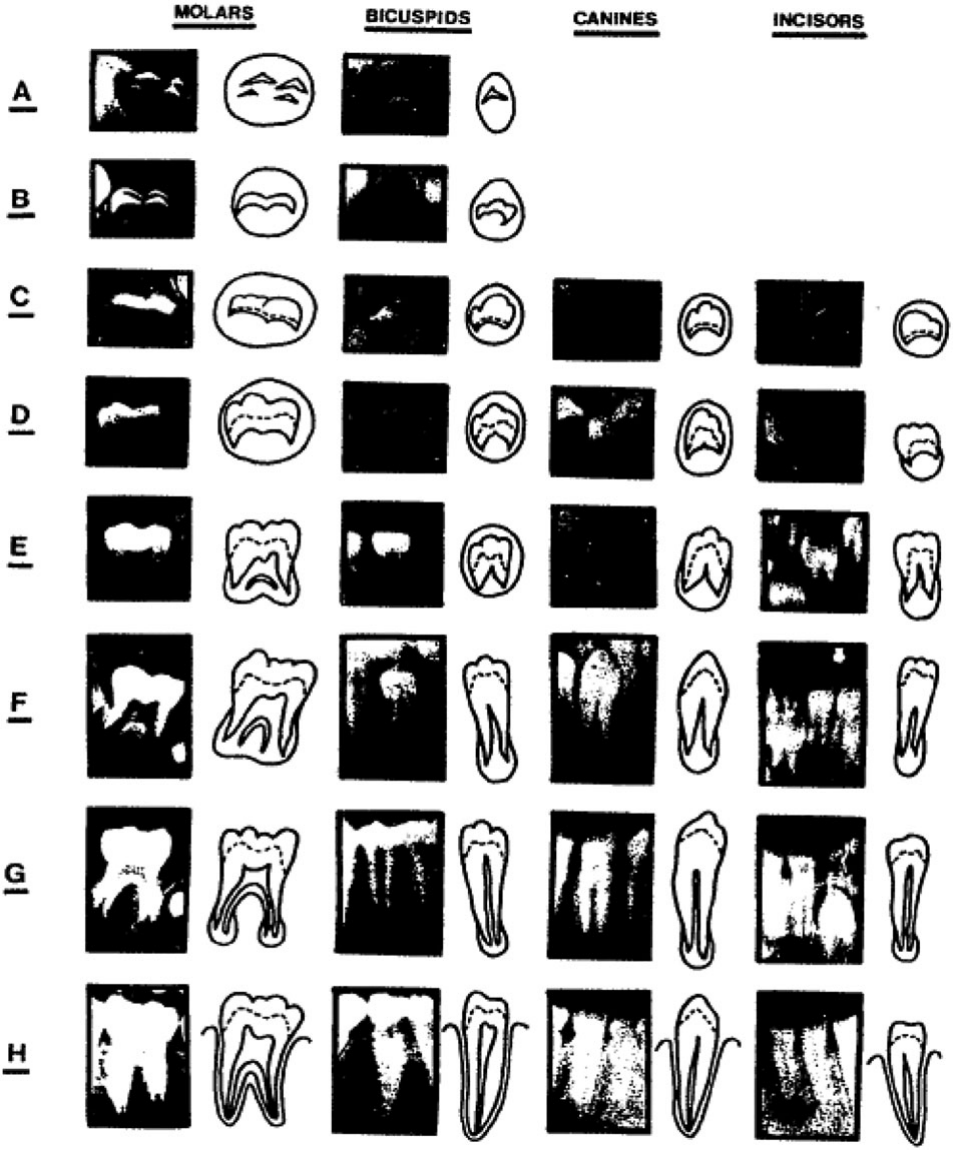
The developmental stages of the permanent dentition (Demirjian et al., 1973)

The dental age estimation was done per Demirjian's method (Demirjian et al., 1973) based on the eight stages (“A” to “H”) of tooth calcification from the tip of the cusp to the closure of the apex as shown in Figure 1. Because this was a subjective method of assessment, two examiners, K. A. and S. H., performed the staging of all seven left mandibular teeth (except the third molar) and converted the score using gender‐specific conversion tables. The same staging was performed a week later by the same set of examiners, K. A. and S.H., and the scores were calculated. This process was performed to minimize errors of misidentification at any stage and to reduce bias in the study. The scores were then added to provide a total maturity score for each individual and was then converted into estimated dental age using gender‐specific standard tables given by Demirjian (Demirjian et al., 1973). The chronological age was determined by subtracting the date of birth and the date on which the panoramic radiograph was taken of the individual.

### 2.3 Statistical analysis

All the relevant data were entered, sorted, and tabulated in Excel (Version:2003, Microsoft, Redmond, USA). The data were statistically analyzed using IBM SPSS (Version 19). The values are represented as the mean ± SD. A paired *t* test was used to determine the significance of differences between dental (Demirjian) and chronological ages. Statistical significance was set at *p* < .05. The degree of inter and intrarater agreement was assessed and calculated using Cohen's kappa statistics.

## 3 RESULTS

**Figure 2:**
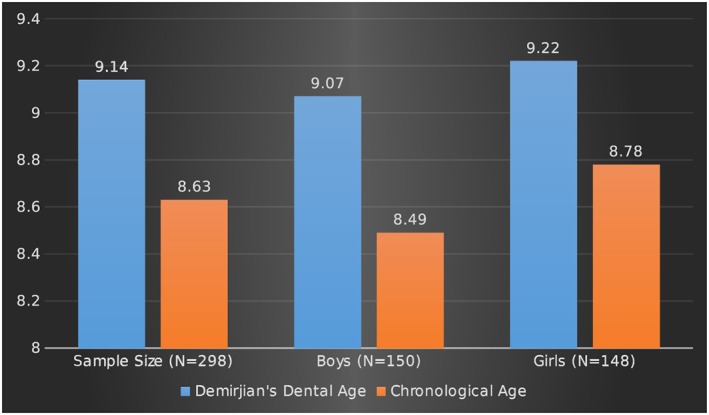
Showing the mean estimated (dental) and chronological age of the sample in years

**Figure 3:**
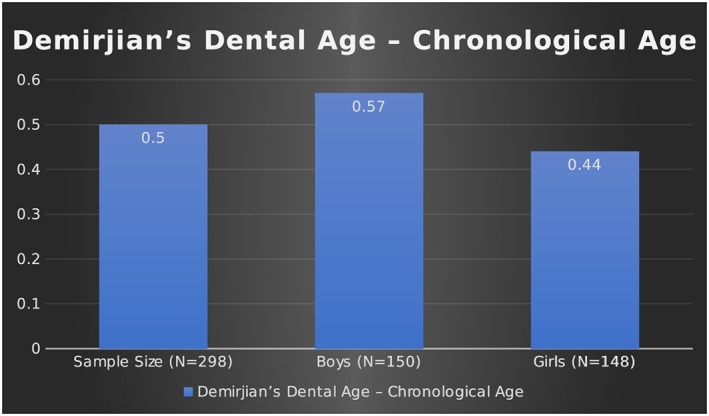
Showing the mean difference between the dental and chronological age of the sample in years

A total of 298 panoramic radiographs for 150 boys and 148 girls between the ages of 3 and 15 years were evaluated in the study. The overall mean dental age of the sample was 9.14 ± 1.94 years, and the overall mean chronological age of the sample was 8.63 ± 2.31 years, as shown in the Table 1 and Figure 2. The mean dental age of the boys assessed in the study was 9.07 ± 1.96 years, and the mean chronological age was 8.49 ± 2.30 years. For girls, the mean dental age was 9.22 ± 1.93 years, and the mean chronological age was 8.78 ± 2.32 years, as shown in the Table 1 and Figure 2. The overall mean difference between the dental and chronological age in the complete sample was 0.50 ± 1.57 years and was found to be statistically significant (*p* < .05). In boys, the mean difference between the dental and chronological age was 0.57 ± 1.48; in girls, it was 0.44 ± 1.66 years. The differences were found to be statistically significant (*p* < .05), as shown in Table 2 and Figure 3. Pearson's correlation revealed a strong positive association between the dental and chronological ages of the complete sample and was found to be statistically significant (*r* = .74; *p* < .05). In boys and girls separately, a strong positive correlation between the dental and chronological ages was found, and it was statistically significant (*r* = .77 and .70, respectively; *p* < .05), as shown in Table 3. The Kappa value for intrarater agreement for the first examiner (KA) was 0.872 (strong) and for the second examiner (SH) was 0.838 (strong). The interrater agreement between examiners K. A. and S. H. was found to be 0.812 (strong).

**Table 1:** Showing the mean estimated (dental) and chronological age of the sample

	Sample size (N = 298)	Boys (N = 150)	Girls (N = 148)
Demirjian's dental age	9.14 ± 1.94	9.07 ± 1.96	9.22 ± 1.93
Mean ± SD (years)			
Chronological age	8.63 ± 2.31	8.49 ± 2.30	8.78 ± 2.32
Mean ± SD (years)			

**Table 2:** Showing the mean difference between the dental and chronological age in the sample

					95% confidence interval				
		Mean (years)	SD	SEM	Lower	Upper	T	df	*p* value
Demirjian's dental age—Chronological age	Sample (*N* = 298)	0.50	1.57	0.091	0.32	0.68	5.576	297	.000
	Boys (*N* = 150)	0.57	1.48	0.121	0.334	0.813	4.739	149	.000
	Girls (*N* = 148)	0.44	1.66	0.136	0.172	0.713	3.234	147	.002

**Table 3:** Showing the Pearson's correlation between the dental and chronological age of the sample

		Correlation (r)	p value
Demirjian's dental age—Chronological age	Sample (*N* = 298)	.74	.000
	Boys (*N* = 150)	.77	.000
	Girls (*N* = 148)	.70	.000

## 4 DISCUSSION

Age estimation by observation of dental structures is a common method that has been used for many years. In 1837, Edwin Saunders was the first person to highlight the role of teeth in age assessment by presenting a pamphlet entitled, “Teeth A Test of Age,” to the English Parliament (Stavrianos et al., 2008). Since then, many methods of age estimation have been proposed, but unfortunately, no universal method exists. This lack may be attributed to the significant amount of variation present in different ethnic groups and populations (Koshy & Tandon, 1998). Demirjian's method is a simple, practical, easily understood method, and it is the most widely used method to estimate age (Stavrianos et al., 2008). The clearly defined criteria in Demirjian's method leaves no room for speculative estimation, thereby making it easy to comprehend and repeat. This method is based on the degree of calcification of tooth structures until the closure of the root apex. Although the eruption pattern and sequence of the teeth can also be used to estimate age that method is considered less reliable, as it is affected by various local and systemic factors (Willems, 2001; Willems, Van Olmen, Spiessens, & Carels, 2001).

This study was undertaken to determine the population‐specific weighted scores needed when Demirjian's method of dental age estimation, which is based on the French‐Canadian population, is applied to the Saudi Arabian population. The data were collected from the five regions of Saudi Arabia, that is, northern, western, eastern, southern, and middle, representing the total population of Saudi Arabia. In the 3‐ to 15‐year‐old age group evaluated in this study, the dental age was found to be higher (overestimated) than the chronological age in both males and females. The obtained dental ages for the children and adolescents of Saudi were found to be higher than those in the French‐Canadian population. In the complete sample of 298 children and adolescents, the mean difference between the dental and chronological ages was 0.50 ± 1.57 years. The difference was 0.57 ± 1.48 years in boys and 0.44 ± 1.66 years in girls. These differences were found to be statistically significant (*p* < .05; Table 2 and Figure 3).

Many studies have been conducted in different populations using Demirjian's method, and their results have varied. Some authors found a high degree of accuracy with their studied population, and some reported either an overestimation or underestimation of dental age compared with chronological age in their studied population. Hagg and Matson (1985) found high accuracy and precision using Demirjian's method on Swedish children. Farah et al. (1999) and Nykanen et al. (1998) studied the Western Australian population and Norwegian children, and they reported a similar result.

An overestimation of dental age over chronological age has been found in many studies, including this one. Koshi and Tandon (1998) reported an overestimation of 3.04 and 2.82 years in males and females, respectively, in South Indian children. In 1999, Liversidge et al. found a dental advancement of 0.51 ± 0.79 years in girls and 0.73 ± 0.73 in boys, which was not significant. Prabhakar, Panda and Raju (2002) reported an average overestimation of 1.20 ± 1.02 years in males and 0.90 ± 0.87 years in females in the child population of Davengere, India. Similarly (Hegde & Sood, 2002; Mani, Naing, John & Samsudin, 2008; Bagic et al., 2008) found dental age to be higher than chronological age in both males and females using Demirjian's method. In the studies performed in the Middle Eastern population, similar results were found. In a study conducted by Al Emran et al. (2008) on Saudi Arabian children between 8.5 and 17 years of age, they found the dental age to be overestimated by 0.3 years for boys and 0.4 years for girls. Baghdadi (2013) reported a mean difference of 0.77 ± 0.85 years for boys and 0.85 ± 0.79 years for girls in Saudi children aged from 4 to 14 years. A study conducted by Qudeimat (2009) on Kuwaiti children aged 3–14 years found an overestimation of dental age by 0.71 ± 1.18 years in boys and 0.67 ± 1.30 years in girls. A recent study performed by Alshihri, Kruger and Tennant (2016) in the Western Saudi Arabian population concluded that girls are 0.059 ± 1.26 years and boys are 0.66 ± 1.14 years ahead of the French‐Canadian children. In this study, we found the dental age to be advanced 0.57 ± 1.48 years in boys and 0.44 ± 1.66 years in girls in the 3–15‐years age group (Table 2 and Figure 3). The difference was found to be significant, indicating that Demirjian's French‐Canadian population does not relate well to the Saudi Arabian population. Hence, a population‐specific weighted scores is needed when applying Demirjian's values to Saudi Arabia's population. This difference that was observed between the dental and chronological ages in various studies, including this one, can be attributed to the universal variations present in ethnicity, culture, sample size, environmental factors, socioeconomic status, nutrition, dietary habits, statistical methods, and subjectivity of the examiner seen in different populations. It was also found in this study that overestimation of age occurs less for girls than for boys. This finding indicates that the females mature much earlier than males, which agrees with the early maturation of skeletal age seen in females. It can also be inferred from the observations that females in Saudi Arabia are more advanced in dental maturity compared with males and that this finding is in accordance with other studies. In this study, a positive and significant correlation was found between the dental and chronological ages in males (*r* = .79) and females (*r* = .70; Table 3). Hagg and Matson in 1985 and Gulati et al. in 1990 also found a high correlation level (*r* = .7–.9 and *r* = .86, respectively) between the chronological and estimated age in children.

Demirjian's method of age estimation is simple, enables reliable standardization, and has good reproducibility and inter/intraexaminer reliability. However, age estimation is an individual‐specific process and most importantly, depends upon the population being considered. No two persons grow and develop at the same rate. According to Nystrom, Ranta, Kataja and Silvola (1988), the differences in overall dental maturity exist not only between nations but also between groups of children in a nation with a relatively homogenous population. Hence, it is imperative that a scoring standard should be based on the results of the studies designed to be used in the same population. The other limitations of this method include the requirement of panoramic X‐rays, which are difficult to obtain and cannot be used to calculate scores in cases of missing teeth. It does not explain agenesis, retarded development, or systemic illness affecting the teeth, and an appreciation of the developmental stages of teeth is quite subjective and cannot be used precisely after 16 years of age. Moreover, this method does not give scores for Stages 1–4 in the case of first molars and central and lateral incisors; hence, exclusion of the individuals below the age of 4.0–4.5 years (Chaillet & Demirjian, 2004; Rózylo‐Kalinowska, Kiworkowa‐Raczkowska, & Kalinowski, 2008).

## 5 CONCLUSIONS

The following conclusions can be drawn from the study:

1. There is variation in Demirjian's standards when they are used for Saudi Arabian children and adolescents. Hence, it is necessary to apply population‐specific weighted scores for more accurate age estimations.
2. In the Saudi Arabian population, the mean difference between the dental and chronological ages was found to be 0.50 ± 1.57 years. In boys, this difference was 0.57 ± 1.48 years, and in girls, it was 0.44 ± 1.66 years.
3. In the studied sample, population‐specific weighted scores (correction factor) to Demirjians's standards were 0.57 ± 1.48 years for boys and 0.44 ± 1.66 years for girls.
4. It is suggested by the authors to use this population‐specific weighted scores when using Demirjian's method of age estimation in Saudi Arabia's children and adolescents.

## AUTHORS CONTRIBUTION

A. A. did the concept, intellectual content, and supervision of the manuscript. K. A. did the data acquisition and analysis. S. H. contributed to the design of the study and clinic work support. A. M. A. did the literature search and review. S. S. A. works on manuscript preparation, editing, and review. M. A. A. did the digital and technical assistance.
